# Variation in the commissioning of specialist weight management services and bariatric surgery across England: Results of a freedom of information‐based mapping exercise across the 42 integrated Care Systems of England

**DOI:** 10.1111/cob.12731

**Published:** 2025-01-19

**Authors:** Maiar Elhariry, Pranav Iyer, Nadya Isack, Bernado Sousa, Pushpa Singh, Sally Abbott, Tom Wiggins, Krishnarajah Nirantharakumar, Srikanth Bellary, Stuart W. Flint, Dimitri J. Pournaras, Jonathan M. Hazlehurst

**Affiliations:** ^1^ College of Medical and Dental Sciences University of Birmingham Birmingham UK; ^2^ Obesity Empowerment Network UK; ^3^ Patient Representative UK; ^4^ Institute of Applied Health Research University of Birmingham Birmingham UK; ^5^ Department of Diabetes and Endocrinology University Hospitals Birmingham NHS Foundation Trust Birmingham UK; ^6^ Specialist Weight Management Service University Hospitals Coventry and Warwickshire NHS Trustd Coventry UK; ^7^ Research Centre for Intelligent Healthcare Coventry University Coventry UK; ^8^ Department of Upper GI Surgery University Hospitals Birmingham Birmingham UK; ^9^ Midlands Health Data Research UK Birmingham UK; ^10^ College of Health and Life Sciences Aston University Birmingham UK; ^11^ School of Psychology University of Leeds Yorkshire UK; ^12^ Scaled Insights, Nexus University of Leeds Yorkshire UK; ^13^ Bristol Weight Management and Bariatric Service, North Bristol NHS Trust Southmead Hospital Bristol UK; ^14^ Centre for Endocrinology, Diabetes and Metabolism Birmingham Health Partners Birmingham UK

**Keywords:** bariatric surgery, commissioning, obesity, specialist weight management, tier 3, tier 4

## Abstract

Specialist weight management services including bariatric surgery are commissioned within regions of England called Integrated Care Systems (ICSs) with eligibility and treatment guidelines determined as part of the National Institute for Health and Care Excellence (NICE) guidance. Reported variation in commissioning and bariatric surgery eligibility criteria has not been previously mapped. Freedom of Information (FOI) requests provide a tool, supported by legislation, to ask questions of public authorities including ICSs such that they must respond accurately. FOIs were sent to all 42 ICSs in England asking 4 questions aiming to establish whether there is variation in the commissioning of specialist weight management services and the eligibility criteria for bariatric surgery across England. Responses were presented descriptively and mapped across England. Responses were received from 41 out of 42 ICSs, with 34 reporting that they provide commissioned medical weight management programmes and 38 funding bariatric surgery. Thirteen reported using criteria that were not compliant with NICE guidance. A large area of the country centred around the East of England does not have a bariatric unit reducing access to care. There is significant geographical variation in the availability of both bariatric and specialist medical weight management services across England, with large portions of the country without local access to a service or no service at all. Where services are available, there is significant inconsistency in eligibility for bariatric surgery despite nationally available guidance.

**What is already known about this subject**

- Bariatric surgery is a life‐changing intervention for people living with obesity associated with significant health gain.
- Within England, the National Institute for Health and Care Excellence (NICE) makes recommendations about which patients are likely to benefit from bariatric surgery.
- Commissioning decisions relating to service provision and treatment criteria are made locally within England in geographical commissioning areas called Integrated Care Systems (ICSs).

**What this study adds**

- Many ICSs apply additional non‐evidence‐based criteria for bariatric surgery not supported by NICE restricting access to treatment.
- Parts of England do not have medical weight management services.
- Parts of England do not have bariatric surgery units.
- Taken together, this leads to significant geographical variation in care.

## 1 INTRODUCTION

The prevalence of adult obesity in England, defined as a body mass index (BMI) ≥ 30 kg/m^2^, is 26%.^1^ Whilst prevention is an important part of national strategy, treatment for people already living with obesity is required to improve quality of life and health outcomes. In addition to the direct and indirect effects of obesity on health and quality of life, there is a significant financial impact with attributable NHS (National Health Service) and wider societal costs in the UK projected to rise to £9.7 billion and £49.9 billion, respectively, by 2050.^2^ The National Institute for Health and Care Excellent (NICE) provides clear guidance for the provision of specialist weight management services across the UK,^3^ however, the extent that services are available is not well defined. Many professional bodies often incorporating patient advocacy groups as well as NHS England have produced recommendations and guidance including on the commissioning of obesity services across the last 10 years to try to improve and standardise service availability and delivery.^4^, ^5^, ^6^ In the UK, obesity treatment is delivered in a four‐tiered system; Tier 1 represents public health interventions, Tier 2 short‐term diet and behaviour interventions typically commissioned by Local Authorities, Tier 3 multidisciplinary team‐led specialist weight management services and Tier 4 bariatric surgery. Both Tier 3 and Tier 4 services fall under the commissioning responsibility of Integrate Care Systems (ICSs).^7^ Prior to bariatric surgery, patients are required to have multidisciplinary support and assessment by a specialist weight management service.^3^ The NICE eligibility criteria for bariatric surgery is a BMI of ≥40 kg/m^2^, or between 35 and 39.9 kg/m^2^ with a significant health condition that could be improved with weight loss (e.g. type 2 diabetes). These criteria have recently been revised with updated guidance published in July 2023 and are now more wide‐ranging and include an extended list of health conditions with clarification that the list is not exhaustive^3^:

- a BMI of ≥40 kg/m^2^, or between 35 and 39.9 kg/m^2^ with a significant health condition that could be improved if they lost weight (examples are not exhaustive but include: cardiovascular disease, hypertension, idiopathic intracranial hypertension, non‐alcoholic fatty liver disease with or without steatohepatitis, obstructive sleep apnoea and type 2 diabetes)
- agree to the necessary long‐term follow‐up after surgery (for example, lifelong annual reviews).

NICE advises a reduction in BMI threshold by 2.5 kg/m^2^ for people with South Asian, Chinese, other Asian, Middle Eastern, Black African or African‐Caribbean family backgrounds. NICE also advises that people with a BMI of 30–34.9 kg/m^2^ and type 2 diabetes diagnosed within the past 10 years can also be considered for bariatric surgery assessment.

The latest iteration of the NICE guideline removes the prior emphasis on patient review in Tier 3 services and instead uses the phrase specialist weight management service. In practical terms, this means that patients could potentially be referred directly to bariatric surgery services rather than first being seen in Tier 3 multidisciplinary team‐led specialist weight management services. Tier 3 services are defined as being multidisciplinary teams typically encompassing a physician, dietitian, psychologist and often with access to physical therapy although definitions vary and outcome data is often limited to short‐term weight loss.^8^ The increased availability of medications for obesity with NICE technology appraisals that must be delivered in Tier 3 (as with Liraglutide [Saxenda])^9^ or within specialist weight management services (but not limited to Tier 3 and 4; as with Semaglutide [Wegovy])^10^ has further increased interest in the availability and commissioning of these specialist services.

Previous work has highlighted the challenges in accurately mapping such services within England; a survey of consultant endocrinologists estimated that by area 60% of England provided a Tier 3 service.^11^ This is reflected in the more recent GIRFT (Getting it Right First Time) Programme National Speciality report for Endocrinology, a large programme of work trying to identify and better understand variations in weight management services. This report showed that only 44% (55) of the 126 hospital trusts visited had Tier 3 services.^12^ There is significant ongoing national work to record and better understand the provision and outcomes of specialist weight management services across the UK including the National Bariatric Surgery Register (NBSR),^13^ NHS England National Obesity Audit^14^ and the Society for Endocrinology National obesity Audit.^15^ The NBSR produces regular reports that focus on outcomes following bariatric surgery and provides useful data to help guide policy. The NHS England National Obesity Audit aims to collect data with a focus on better understanding treatment access and coverage and provision of services^14^ although a number of large centres have been unable to engage with it due to technical challenges of hospital‐based electronic systems being unable to provide data to the required Community Services Data Set (CSDS). Thus, it is clear therefore that an accurate picture of available services is required to support policy development and commissioning strategy. Within England, decisions on health service commissioning are made by ICSs. These ICSs have budgetary responsibility for most commissioning decisions with some specialised commissioning responsibility held centrally by NHS England. ICSs make commissioning decisions for how health care is delivered and funded within their ICS. Until 2017, bariatric surgery and specialist weight management services had been commissioned centrally^6^ when commissioning responsibility was transferred to clinical commissioning groups (CCGs), which were predecessors of ICSs.

The Freedom of Information (FOI) act (FOIA) 2000 FOIA is increasingly recognised as a useful tool for social science research and those working on policy. Due to the legal requirement to respond, using FOI requests should result in a more complete data set than equivalent work with surveys or enquiries.^16^ Work mapping Attention‐Deficit/Hyperactivity Disorder services previously published highlighted the relative efficacy of FOI compared to emailed surveys with 80% and 9% responses, respectively.^17^

Given the commissioning responsibility of ICSs, the primary aims were to (1) determine whether they commission bariatric surgery and specialist weight management services, (2) to identify what criteria are used for eligibility for bariatric surgery services across England, and (3) determine whether these criteria were compliant with the NICE recommendations.

## 2 METHODS

Authors JMH, PI and ME drafted 4 questions to form the basis of the FOI requests. The questions were further refined with patient involvement guidance from author NI, and further review from experts in the field, authors SA and DP. The final wording of the 4 questions, which were sent to Integrated Care Systems (ICSs) via FOI requests on 26.7.23 was:

1. Do you fund bariatric surgery for people living in your ICS?
2. Please list all the current criteria that are used within your ICS for funding decisions regarding bariatric surgery.
3. Is there a commissioned/designated bariatric surgery service located within your ICS catchment? If not, where are people requiring bariatric surgery within your ICS typically referred?
4. Do you commission a Tier 3 specialist weight management service? Is it located within your ICS catchment? If not, where are people within your ICS requiring Tier 3 weight management services typically referred?

These 4 questions were sent to the FOI office of each individual ICS. The responses were then recorded. Obtaining the required information followed an iterative process with some ICSs signposting to already available information, NICE guidance or the prior national service specification. To assess the eligibility criteria provided as being NICE compliant criteria had to either explicitly mention that they were in accordance with NICE criteria or the stated criteria had to be consistent with NICE and in particular had to include eligibility of BM ≥40 kg/m^2^ or ≥ 35 kg/m^2^ with related health conditions. The FOI requests were sent before the latest iteration of NICE guideline CG189 although the BMI cut‐offs used to assess bariatric surgery have not changed across the latest iteration.

Response rate and completeness as well as the answers to the 4 questions were recorded. This information was subsequently presented using descriptive statistics, and maps were generated using a software licence purchased from GB Maps.^18^

## 3 RESULTS

Out of the 42 ICSs in England, 41 (98%) responded to our FOI requests. Overall, ICS representatives responded to the majority of the questions. A number of ICSs provided a link to their respective websites in response to some of the questions, indicating that the data is accessible to the public already in accordance with Section 21 of the FOIA.

### 3.1 Do you fund bariatric surgery for people living in your ICS?

Most (90%; 38/41) of the ICSs who responded to this question provided clear information that they fund bariatric surgery. One ICS clearly stated that although they do not have a bariatric surgery centre, they fund bariatric surgery for their patient cohort within other ICSs. Of the 3 ICSs who did not clearly answer yes to this question, one stated that patients could be referred elsewhere but made no comment on funding, whilst two ICSs stated (incorrectly) that bariatric surgery was commissioned by NHS England and suggested we contacted them.

Five of the 38 (14%) ICSs confirmed that they fund bariatric surgery, and provided further detail beyond what was asked in response to this question. Two reported that they fund bariatric surgery only if certain criteria are met without expanding on the criteria, and one ICS added a link to the criteria required to be eligible for funding. ICSs stated which weight management services, in terms of Tiers, they provide funding for and recommended contacting the providers for further information. One ICS added that it funds bariatric surgery “via its value‐based commissioning policy”.

### 3.2 Please list all the current criteria that are used within your ICS for funding decisions regarding bariatric surgery

**FIGURE 1:**
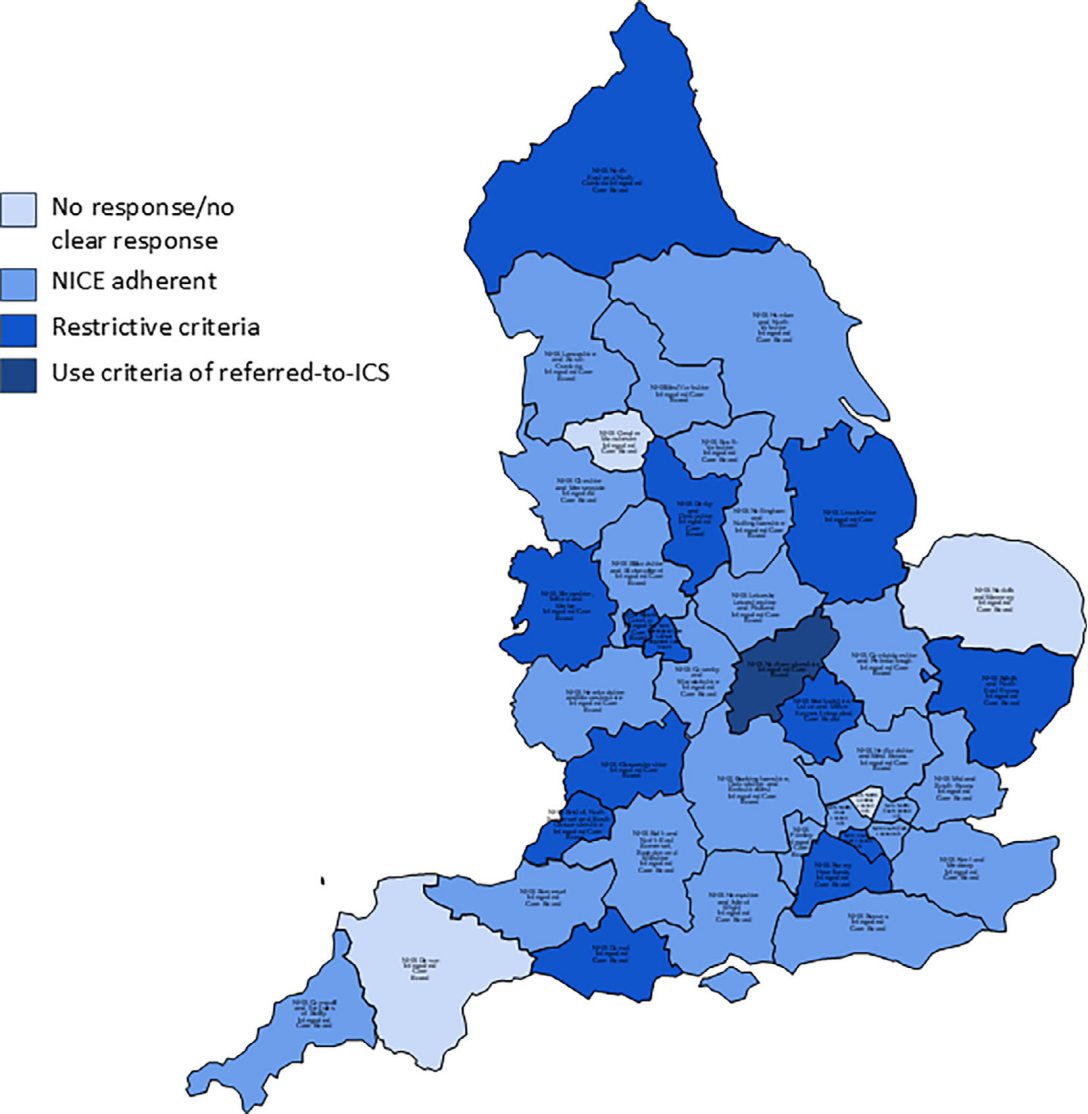
Map of ICSs reporting eligibility for bariatric surgery and their concordance with NICE guidance.

Out of the 38 ICSs that report that they fund bariatric surgery, 24 (63%) either directly quoted that they follow NICE Clinical Guideline 189 or cited the criteria from the guidance (Table 1; Figure 1). Thirteen (34%) out of the 38 ICSs that report funding bariatric surgery had more restrictive criteria to determine patients' eligibility for bariatric surgery referral than those set by NICE. Those included higher BMI thresholds (e.g. ≥ 50 kg/m^2^) restriction in the health‐related conditions that qualify for the intervention (e.g. the lower BMI cut‐ff of 35 kg/m^2^ only applicable to people with type 2 diabetes) and additional requirements not set by NICE (including minimal duration of obesity). One ICS stated that the eligibility criteria depend on the ICS the patients seek referral to as they do not provide bariatric surgery locally within the ICS. The ICS that redirected question 1 to NHS England included criteria that met those set by the NICE guidelines but were not included in this analysis as they did not explicitly declare they fund bariatric surgery.

**TABLE 1:** Breakdown of provided bariatric surgery eligibility criteria supplied by ICSs compliant with NICE.

ICSs purporting to be NICE compliant, *n* (% of total ICSs)	24 (63%)
Age cut off	
ICSs providing this information	6
Age ≥ 18 years	6
BMI cut offs	
ICSs providing this information	19
BMI ≥35 kg/m^2^ with comorbidities	19
BMI ≥40 kg/m^2^	19
Comorbidities: required for surgery at the lower BMI cut off ≥ 35 kg/m^2^	
ICSs providing examples of comorbidities	14
ICSs not expanding on comorbidities	7
Need for commitment to long‐term follow‐up	
ICSs providing this information	10

Abbreviations: BMI, body mass index; ICS, Integrated Care System; NICE, National Institute of Health and Care Excellence.

From the 24 ICSs that were consistent with NICE recommendations, 21 either included non‐exhaustive examples of comorbidities with phrases including “such as” or “for example” or “significant comorbidities” or simply mirrored the NICE guidance. However, three ICSs that here were classified as NICE compliant did provide a clear list of required comorbidities for eligibility with a BMI ≥35 kg/m^2^. Ten ICSs expressly stated the need for long‐term follow‐up. The geographical distribution of ICSs across England reporting eligibility criteria for bariatric surgery is presented (Figure 1).

Out of the 38 ICSs that provided clear confirmation that they fund bariatric surgery, 13 had more restrictive eligibility criteria with more requirements than included in the NICE guideline. All 13 acknowledged that their criteria are more restrictive. One ICS that did not have its own bariatric unit reported using the eligibility criteria of the out‐of‐area ICSs that patients could be referred to for surgery. Of the four ICSs that did not provide a clear statement confirming funding bariatric surgery, one did not respond at all to the FOIs, one provided no response to this question, one signposted to generic information on the NHS website and one incorrectly signposted to NHS England. These four ICSs have been classified as “No response/No clear response” (Figure 1). Of the 13 ICSs with restrictive criteria, the duration of obesity severity was one such restrictive criterion with four ICSs stating that patients referred for bariatric surgery must have “morbid/severe obesity for at least five years” with another ICS mandated that to be referred, a patient must have “a documented record of having a BMI of 40 kg/m^2^ or over for at least 24 months”. Three ICSs mentioned higher BMI cut‐off for surgery compared to the recommended BMI of ≥40 kg/m^2^ by the NICE guideline—with three ICSs setting a minimum BMI of 50 kg/m^2^ and one ICS setting a minimum BMI of 60 kg/m^2^–7 ICSs included minimum durations ranging from engagement with a Tier 3 weight management service or other non‐surgical options for a minimum set duration (Table 2). Other restrictive criteria included: a more limited set of comorbidities in those with a BMI ≥35 kg/m^2^, and mandatory preoperative weight loss.

**TABLE 2:** Breakdown of responses from ICSs with restrictive eligibility criteria for bariatric surgery.

Restrictive criteria	Number of ICS with more restrictive criteria
Total ICSs with restrictive criteria	13
A minimum duration of moderate/severe obesity for or a BMI of ≥40 kg/m^2^	5
Engagement with a Tier 3 weight management service or other non‐surgical options for a minimum set duration	12 months: 2
	12–24 or 24 months: 4
Restrictive list of comorbidities at lower BMI threshold	4
Mandatory pre‐operative weight loss	1
A higher BMI cut‐off	BMI ≥50 kg/m^2^: 3
	BMI ≥60 kg/m^2^: 1

*Note*: In compliance with the NICE guidance,^3^, ^29^ a BMI of ≥30 kg/m^2^ with recent‐onset type 2 diabetes and accessing Tier 3 services or attempted weight loss interventions before considering surgery should be considered for an assessment, only one ICS included this as an eligibility option.

Abbreviations: BMI, body mass index; ICS, Integrated Care S.

### 3.3 Is there a commissioned/designated bariatric surgery service located within your ICS catchment? If not, where are people requiring bariatric surgery within your ICS typically referred?

**FIGURE 2:**
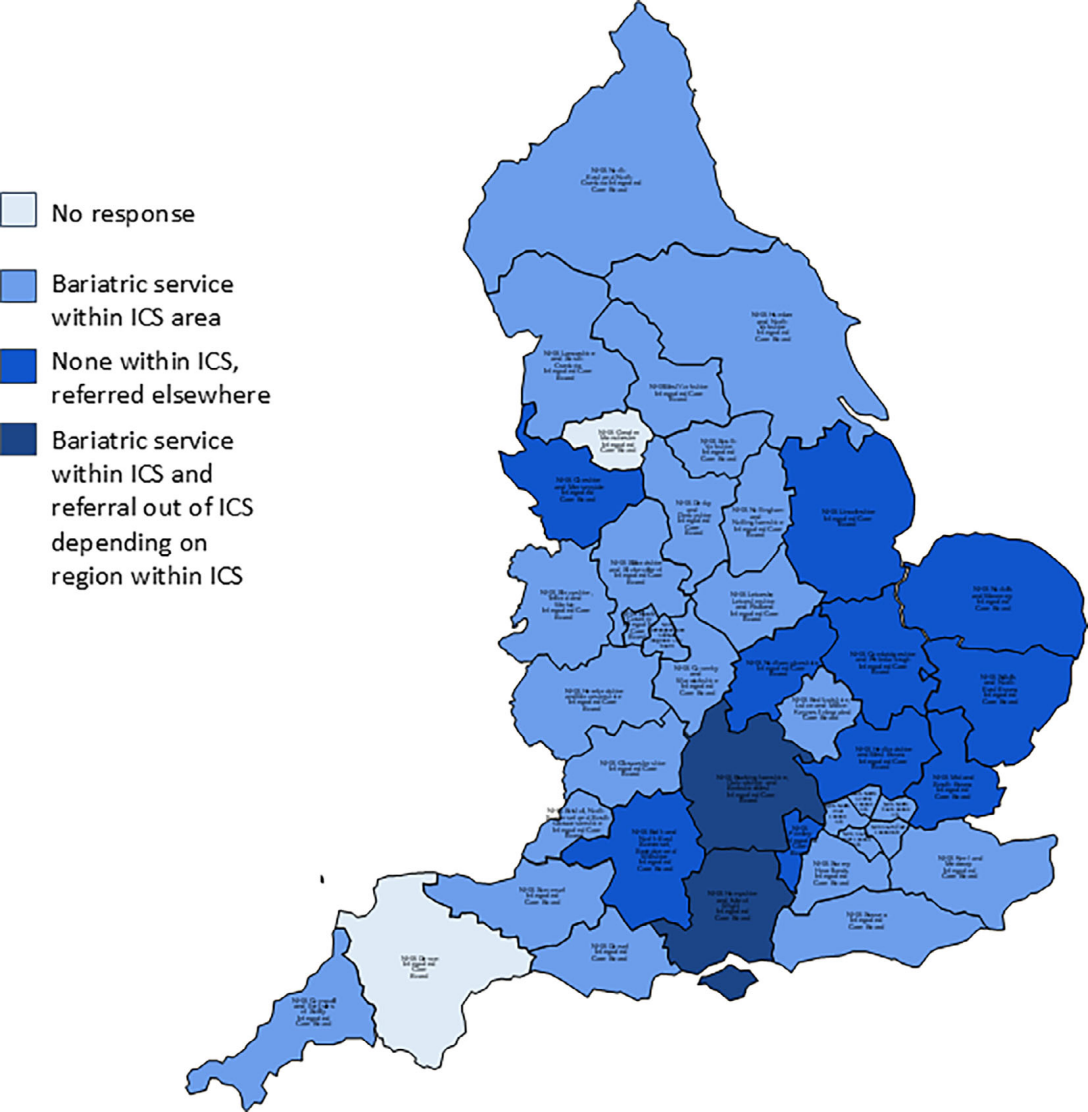
Map of ICSs reporting locations of bariatric surgery centres in relation to their geographical ICS catchment.

Of the 41 ICSs that responded to the initial FOI request, 30 of 41 (73%) stated that they do have a commissioned/designated bariatric surgery service within their ICS catchment (Figure 2). Of these two ICSs reported that it depends on where in the region as some patients were also referred out of ICS to more local providers. Ten of 41 (24%) stated an absence of service within the ICS catchment area and provided alternative sites for referral for commissioned/designated bariatric surgery. One ICS responded stating incorrectly, that “Tier 4 weight management bariatric surgery is specialised commissioning and therefore the commissioning responsibility of NHS England” and as such directed the research team to NHS England.

### 3.4 Do you commission a Tier 3 specialist weight management service? Is it located within your ICS catchment? If not, where are people within your ICS requiring Tier 3 weight management services typically referred?

**FIGURE 3:**
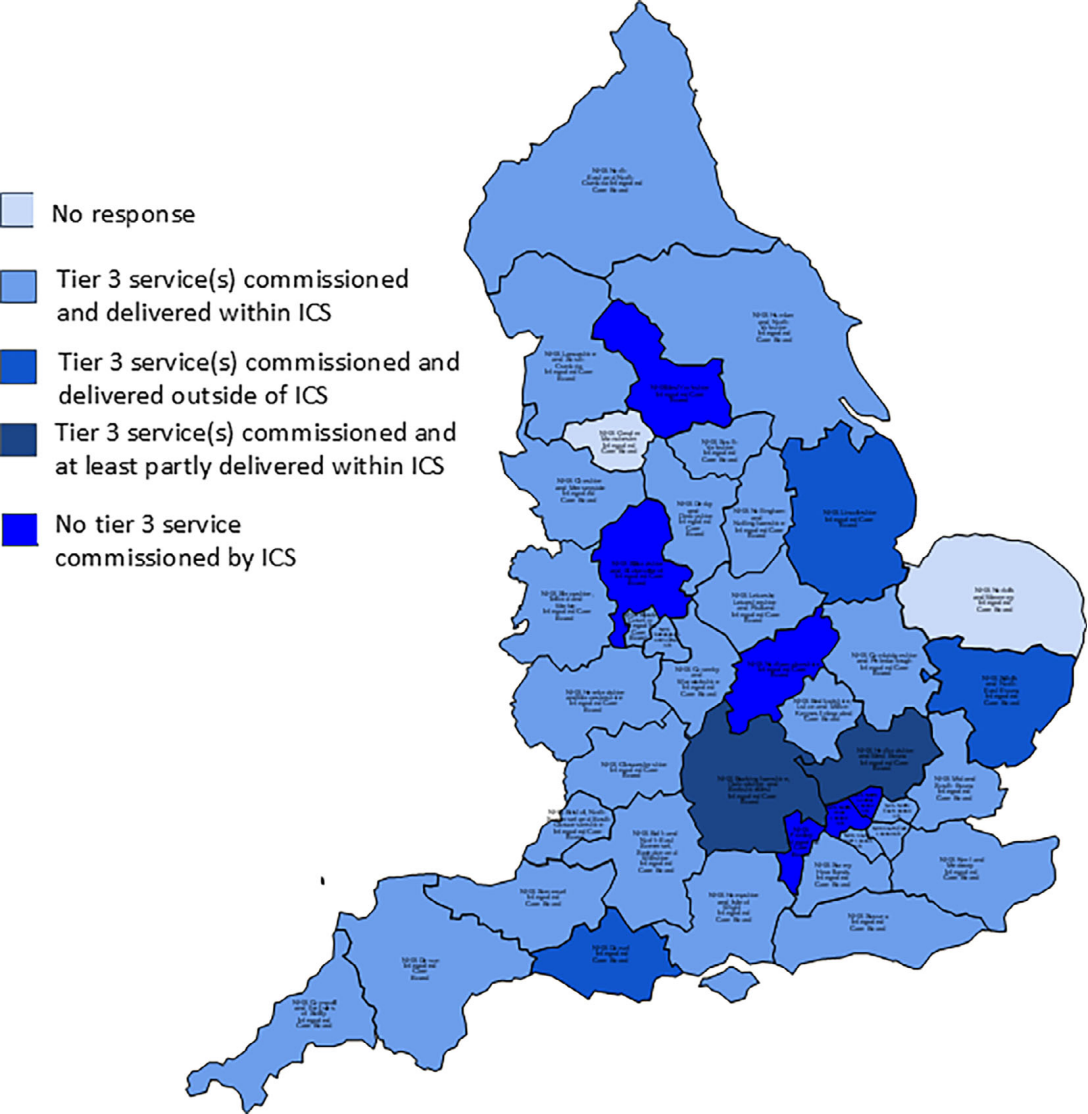
Map of ICSs reporting whether they commission Tier 3 weight management centres in relation to their geographical ICS catchment.

Thirty‐four of 41 (83%) ICSs that provided some level of response to the FOI request stated that they commissioned a tier 3 specialist weight management service (Figure 3). However, of these, two ICSs report significant variation in provision within their ICS area including referral out of the area for some patients and one ICS reports that not all patients within their ICS catchment have access to a Tier 3 but that they are scoping further provision and one ICS accepted that whilst a service was commissioned, the provision was limited and not consistent with what it recognises would be expected of NICE guidance for a specialist service. One ICS commissioned a service that was entirely delivered outside of the ICS catchment. One ICS reported a service that was jointly commissioned with the local authority and was included in this analysis as ICS commissioned. Of the 34 that reported commissioning such services, only 29 (71%) provided services entirely within their ICS catchment. Of the five ICSs clearly responding “No” to this question, one ICS reported ongoing work to scope out the provision of a service and two ICSs commented further that patients could be referred elsewhere. One additional ICS that did not respond “Yes” or “No” provided information highlighting Local Authority commissioned services and therefore should have answered “No” as it did not provide any information that it was jointly commissioned, it did however report that it was working on setting up a service. One ICS that responded to other questions within the FOI responses did not respond to this question.

## 4 DISCUSSION

This is the first published study using data generated from FOI requests to systematically map ICS commissioning of bariatric surgery and specialist weight management services across England. The current study findings highlight that whilst most ICSs report funding bariatric surgery, there is considerable variation in the eligibility criteria that patients must meet to be considered for NHS funded bariatric surgery. Of the 41 ICSs who responded to our FOI requests, 38 confirmed that they fund bariatric surgery, and of these, only 24 reported either being compliant with NICE guidance CG189 or presented criteria that were in accordance with this guidance. One ICS, without a bariatric unit, reported using the criteria of the ICS that its patients were referred to. Thirteen ICSs apply more restrictive criteria to access bariatric surgery such as restrictive BMIs, duration of comorbidities and obesity or a requirement for preoperative weight loss. This is concerning as this highlights that 31% of ICSs are not following NICE recommendations and thus, evidence‐based practice.

Since 2017, the commissioning of bariatric surgery has been devolved to localities, initially clinical commissioning groups, and more recently, ICSs.^6^ Within this service specification, the criteria for the transfer of this funding responsibility to localities is clear. One of the unintended findings from this study was that two ICSs reported that bariatric surgery is centrally commissioned despite that the commissioning responsibility was transferred to the CCGs (now ICSs) since 2017.^6^ Bariatric surgery is an effective treatment for people living with obesity and results in improved QoL,^19^ reduction in cardiovascular mortality,^20^ reduction in all‐cause mortality^21^ as well as improvement outcomes in patients living with type 2 diabetes.^22^

Our data shows that commissioning of specialist weight management services across England was limited with only 83% of ICSs reporting commissioning these services; however, only 71% commissioned services delivered within the ICS geographical catchment. Our data about medical services reflects data from the recent GIRFT report^12^ with significant areas of the country without access to such services. There is a possibility that we have not identified services that are commissioned by Local Authorities rather than ICSs. The authors read with sadness about the recent closure of one such local authority‐commissioned service which has been held in high regard both nationally and locally by the patients it supports.^23^ Given the advent of increasingly effective interventions including meal replacement products and medications to augment lifestyle modification, our finding that a significant number of ICSs across England are not commissioning specialist services lets down people living with obesity. One of the unanticipated findings was that one ICS that does not commission a Tier 3 service reported that “*Tier 3 services are not typically consultant led and therefore the patient “right to choose” does not apply and in such circumstances service providers outside the ICS are not obliged to accept”*. This is an important point about the provision and funding of care for patients with obesity across different ICSs and if accepted should prompt ICSs to ensure local services are commissioned. Current waiting times across obesity services are at an all‐time high and there is a significant mismatch between the demand and provision of high‐quality MDT‐led care, this has culminated in 1 in 6 ICSs having closed their lists to new weight management referrals.^24^ There are ongoing pilot studies rolled out in the community which include digitally delivered care and/or increased availability of medications for obesity.^25^ They are a welcome step to give more patients access to treatment. However, it is too early to predict if these systems in place will widen access or even potentially exacerbate health inequalities given the significant geographical variation in service provision. The current study did not gather data on treatments offered within Tier 3 services which will be needed in response to NICE approvals for obesity medications.

This study highlighted that a significant proportion (31%) of ICSs apply more restrictive criteria to access bariatric surgery. As such, nearly a third of ICSs are non‐compliant with NICE guidance and evidence‐based practice. Understanding the barriers and impact of ICSs applying more restrictive criteria than NICE guidance on both people living with obesity and healthcare systems is needed. The findings of this study are important for policymakers and highlight the need for a nationally coordinated strategy for obesity treatment provision. Policymakers, NICE and the ICSs that are non‐compliant with NICE guidance should consider why regional variation is applied in the case of obesity but is not for other long‐term health outcomes where national guidance is applied, avoiding potential inequalities driven by local decision‐making. Regional variations in the application of NICE guidance would not be accepted for other long‐term health conditions. This decision may reflect the ingrained stigma towards obesity that has been consistently highlighted in healthcare.^26^, ^27^

One of the key limitations was that our study focused on bariatric surgery provision in England and did not extend our work to Wales, Scotland and Northern Ireland or more internationally. The decision to focus on England in the current study was made pragmatically to assess the responses of the ICSs which were created in 2022 as a move away from the prior CCGs.^28^ We are aware of significant variation in care and service availability across the wider UK but this work was not designed to assess this important point. This work focused on the ICS commissioning of clinical services and an unintended consequence is that we may not have picked up on non‐commissioned specialist weight management services or services that are commissioned by local health authorities. Since the responsibility for commissioning sits with ICSs, this is likely to represent a small proportion of services. A further limitation is the reliance on the response quality to the FOI requests, although it is a legal requirement that responses to FOI requests are accurate. Despite these limitations, the potential implications of the findings presented in this study highlight the need to ensure there is an accurate picture of the commissioning of bariatric surgery and weight management services in England, and thus, we welcome further collaboration that confirms the commissioning models employed across the country.

The current work highlights significant inequity with access to effective treatment, whether that be geographically or by locally applied none‐evidence‐based restrictive criteria for bariatric surgery. At the centre of this issue are people living with obesity, unable to access care, many of whom have progressive obesity‐associated medical conditions or unable to access other treatments including orthopaedic surgery or fertility treatment who are currently let down by the current lack of effective available local services.

## AUTHOR CONTRIBUTIONS

JMH, DP and TW conceived the study with further input in its design by ME, PI, NI, KN, PS and SA. NI, BS, PS, SB, SA, KN, SB, DP and SF provided critical input into the analysis and presentation of the data. ME, PI and JMH wrote the initial manuscript and analysed the data. All authors were involved in writing the paper and had final approval of the submitted and published versions.

## FUNDING INFORMATION

This work was supported by a wider NIHR West Mids Clinical Research Network I&I award to KN, SB and JH as well as an NIHR West Mids Clinical Research Network Research Scholarship to JH.

## CONFLICT OF INTEREST STATEMENT

ME and PI have no conflicts of interest. NI declares being a trustee of the Obesity Empowerment Network and an active patient advocate. BS declares being an active patient advocate. PS has no conflicts of interest. SA declares research support from Johnson & Johnson as well as the British Dietetic Association as well as consulting fees from AstraZeneca and Johnson & Johnson and ICE creates as well as support for attending educational meetings from Novo Nordisk and the British Dietetic Association as well as committee‐level membership of British Obesity and Metabolic Surgery Society and British Dietetic Association Obesity Specialist Group and Obesity Management Collaborative, UK. TW has no conflicts of interest. KN declares grants from NIHR, UKRI/MRC, Kennedy Trust for Rheumatology Research, Health Data Research UK, Wellcome Trust, European Regional Development Fund, Institute for Global Innovation, Boehringer Ingelheim, Action Against Macular Degeneration Charity, Midlands Neuroscience Teaching and Development Funds, South Asian Health Foundation, Vifor Pharma, College of Police, and CSL Behring as well as consultancy fees from BI, Sanofi, CEGEDIM and MSD and leadership roles within NICST, a charity and OpenClinical, a Social Enterprise. SB declares speaker fees and AstraZeneca, Boehringer Ingelheim, NovoNordisk and Eli Lilly and received research funds from AstraZeneca and Bayer. SWF declares grants from NIHR, Office of Health Improvement and Disparities, Doncaster Council and Novo Nordisk as well as support for attendance at meetings or travel from UK Parliament, Johnson and Johnson, Novo Nordisk, Devon NHS Integrate Care Service and Safefood. DJP declares consulting fees from Johnson & Johnson, Novo Nordisk, Pfizer, GSK, and Medtronic as well as honoraria from Johnson & Johnson, Medtronic, Sandoz and Novo Nordisk. JMH declares grant funding from NIHR and previous advisory feed and speaking honoraria from Novo Nordisk as well as support for attendance at meetings from Novo Nordisk and Eli Lilly as well as being a committee member of Obesity Management Collaborative UK.
